# In Vivo Intraocular Lens Thickness Measurement and Power Estimation Using Optical Coherence Tomography

**DOI:** 10.18502/jovr.v17i3.11572

**Published:** 2022-08-15

**Authors:** Ehsan Barzanouni, Diba Idani, Farideh Sharifipour

**Affiliations:** ^1^Ophthalmic Research Center, Research Institute for Ophthalmology and Vision Science, Shahid Beheshti University of Medical Sciences, Tehran, Iran; ^2^Department of Ophthalmology, School of Medicine, Shahid Beheshti University of Medical Sciences, Tehran, Iran; ^3^School of Medicine, Shahid Beheshti University of Medical Sciences, Tehran.

**Keywords:** Anterior Segment Optical Coherence Tomography, AS-OCT, Intraocular Lens, IOL, IOL Thickness

## Abstract

**Purpose:**

To estimate the power of an implanted intraocular lens (IOL) by measuring IOL thickness using anterior segment optical coherence tomography (AS-OCT) and to assess the repeatability of measurements.

**Methods:**

Ninety-seven eyes were studied one month after uneventful phacoemulsification within the bag Acrysof SA60AT IOL implantation (range +11 to +35). All eyes had postoperative refraction of ± 0.5 D of target refraction. AS-OCT was used to measure the central thickness of the IOL. Correlation between labelled IOL power and central IOL thickness as well as the measure of repeatability, for example, intraclass correlation coefficient (ICC), were evaluated. IOL thicknesses were also calculated using a formula and compared with AS-OCT derived measurements.

**Results:**

IOL thickness correlated significantly with labelled IOL power (R^2^ = 0.985, *P* < 0.001). The regression equation (IOL Power = [0.04 × IOL thickness in micron] – 7.56) indicates 25 microns of central IOL thickness change per 1D power change. Over the studied range, IOL power could be estimated with a precision of 0.85 ± 0.02 D (95% confidence interval: 0.83–0.94D). ICC for repeated measurements was 0.999. There was a significant correlation between calculated and measured (AS-OCT) IOL thickness (R^2^ = 0.984, *P* < 0.001).

**Conclusion:**

Central IOL thickness measurements with the AS-OCT are highly repeatable and closely correlated with the labelled IOL power, which can predict the IOL power with ± 0.85 D from the actual power. This method can be helpful in cases of postoperative IOL surprise.

##  INTRODUCTION

Cataract removal with intraocular lens (IOL) implantation is one of the most frequently performed ophthalmic surgeries. Microsurgical techniques, improved IOL material and designs, sophisticated biometry methods, and advanced IOL power calculation formulas have altered the role of cataract surgery even as a refractive surgery, where in addition to removing opaque crystalline lens also corrects any preexisting ametropia. The accuracy of the ocular measurements and IOL calculation, as well as selection of the appropriate biometric formula, are the main factors in achieving the desired postoperative refractive results.^[1]^ However, despite all these measures, refractive surprise might happen as a result of transcription errors, wrong patient biometry, wrong IOL selection, changes in planned procedure, incorrect IOL brought into the theatre, left/right eye selection errors, communication errors, and positive/negative IOL power errors.^[2]^ In rare cases, incorrect IOL labelling might be the cause.^[3,4,5]^ However, in 25–38% of the cases, the cause of refractive surprises remains unknown.^[2]^ Although there is a need to calculate the power of an implanted IOL, currently there is no established method, and knowledge of implanted IOL power is only restricted to the medical records of the patients.

The introduction and evolution of imaging techniques especially optical coherence tomography (OCT) has made it possible to image the ocular structures with micron-level precision. OCT measurements have been shown to highly correlate with real values, making it an ideal method for evaluating anterior segment structures.^[6,7]^ Scheimpflug imaging has been used for central IOL thickness measurement and in vivo calculation of IOL power.^[8]^ This study was conducted to assess the correlation of IOL thickness measured by anterior segment OCT (AS-OCT) with the actual power of implanted IOL to calculate the power of an unknown IOL.

**Figure 1:**
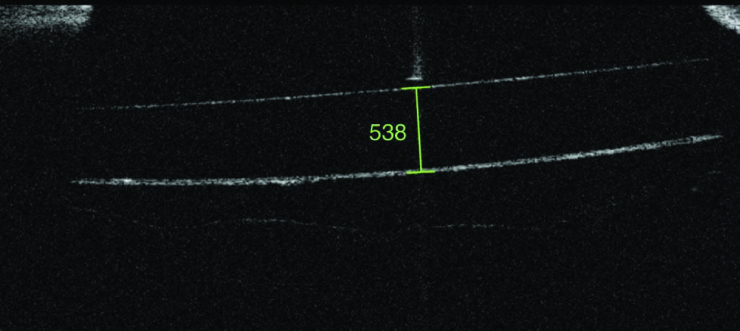
Representative image of IOL thickness measurement method using anterior segment OCT.

**Figure 2:**
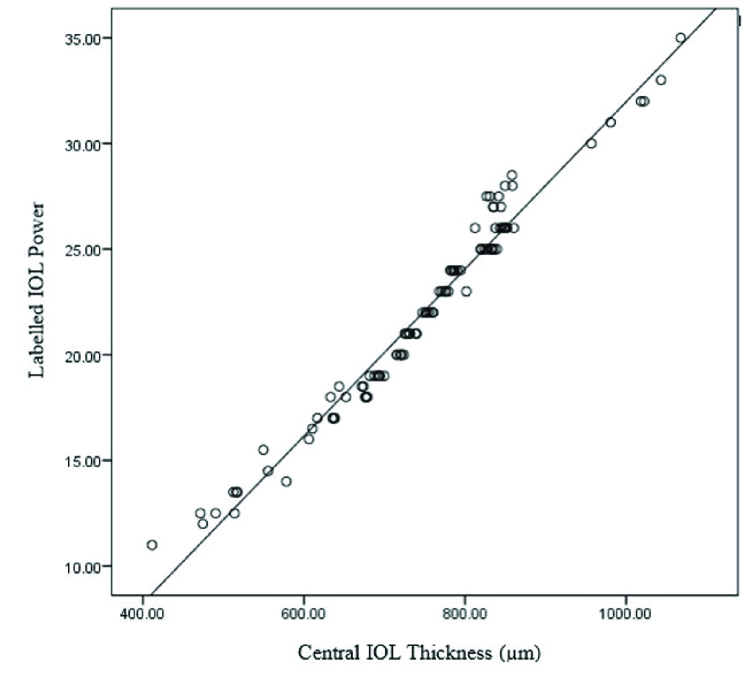
Plot of IOL thickness (microns) against labelled IOL power (D).

**Figure 3:**
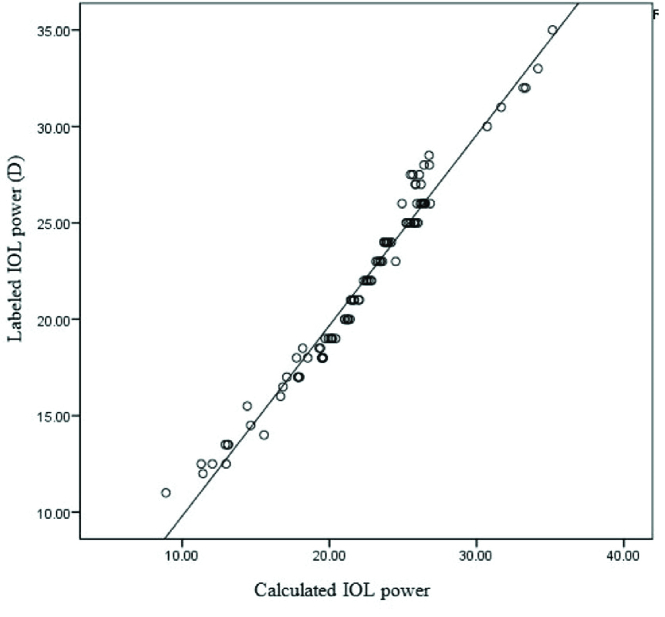
Plot of labelled IOL power against IOL power calculated by the measured regression equation showing significant correlation.

**Figure 4:**
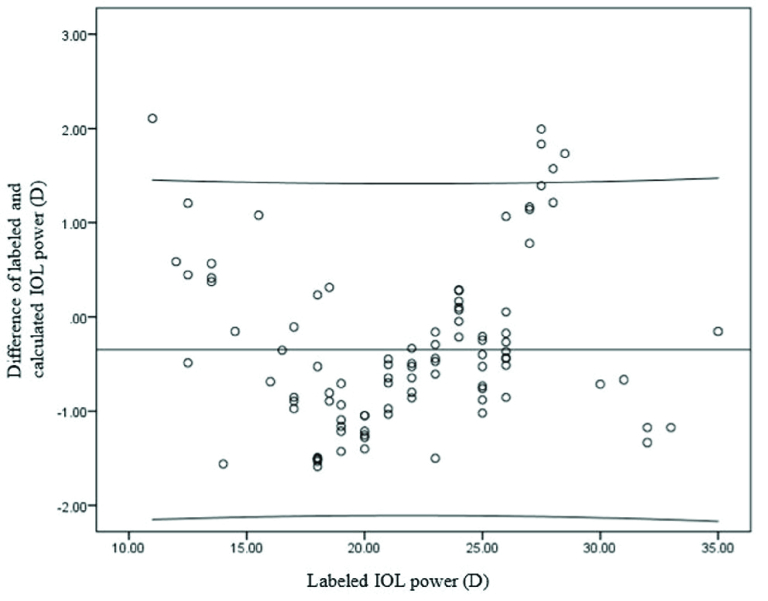
Plot of difference of labeled and calculated IOL powers against actual IOL power. The majority of labelled IOL powers lie within Mean ± SD of difference of labeled and calculated IOL powers.

**Figure 5:**
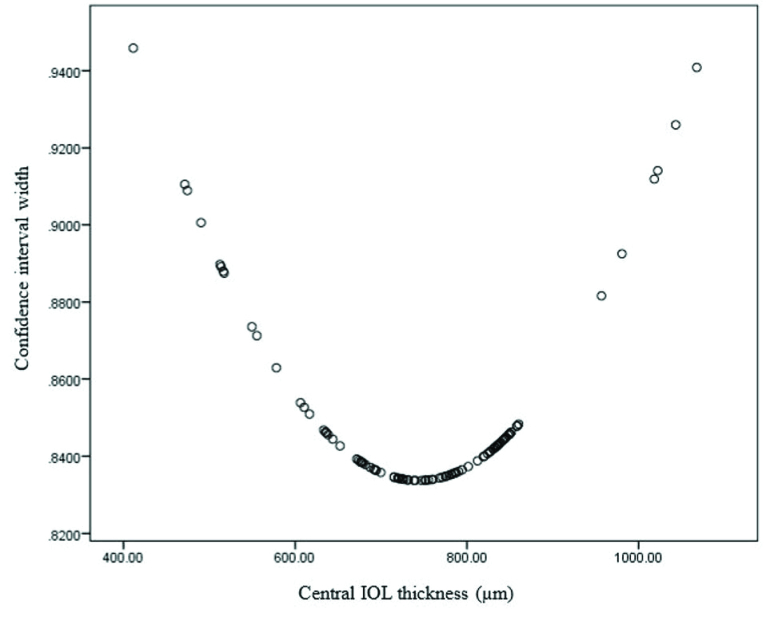
Plot of IOL thickness against confidence Interval (CI) width showing that 95% CI is the narrowest for IOL thicknesses between 600 and 900 microns and becomes wider outside this range.

##  METHODS

This prospective study was performed at a private clinic. The study protocol adhered to the tenets of the Declaration of Helsinki. Informed consent was obtained from all patients and the test was performed free of charge.

The study included consecutive patients who underwent uneventful phacoemulsification within the bag Acrysof SA60AT IOL (Alcon) implantation. At postoperative month one, patients with uncorrected visual acuity of 20/25 or better who manifested refraction within ± 0.5 D spherical equivalent of target refraction were enrolled in the study. Patients with corneal opacity precluding high-quality images, history of trauma or anterior segment diseases causing pupil abnormality, IOL decentration, iridodonesis, pseudophacodonesis, and IOL tilt were excluded.

Acrysof SA60AT IOL is a monofocal foldable, single-piece posterior chamber acrylic lens with an asymmetric biconvex 6 mm optic, overall length of 13 mm, and a refractive index of 1.55. Available powers range from 6.0 to 30.0 D in 0.5 D increments and from 30 to 40 in 1D increments.

### Optical Coherence Tomography Measurements

OCT scans were performed using anterior segment module, (Topcon 3D OCT-1000 Topcon Corporation, Tokyo, Japan) after pupillary dilation. The scan line was centered on the IOL along the horizontal line and three images were captured. The presence of a reflex saturation beam indicated the perpendicularity of the IOL to the scanning beam [Figure 1]. Lens thickness was measured using a built-in caliper from the anterior to the posterior surface of IOL at the point of greatest convexity. On each scan, measurements were done by two observers (FS, DI) masked to the IOL power. The average of the three closest measurements was used for the analyses. Scans with a lens tilt, motion artefact, and adhesion of posterior capsule to the IOL were discarded.

### Statistical Analysis 

Statistical analysis was performed using the SPSS software version 18.0 (SPSS Inc., Chicago, USA). The intra- and interobserver repeatability of IOL thickness measurements were assessed using intraclass correlation coefficient (ICC). Correlation between IOL thickness and labelled IOL power was evaluated by Pearson's correlation and linear regression analysis; 95% confidence intervals (CI) were calculated for every IOL prediction based on thickness measurements. In addition, we calculated central IOL thickness using the formula proposed by Naeser et al^[9]^ for each IOL power and compared them with OCT-derived thickness measurements. 


$$T=E+2\times \left(\left|\left(n2-1.336\right)\times \frac{1000}{1/2\times P}\right|\right.\left.-\sqrt{{\left(\left(n2-1.336\right)\times \frac{1000}{1/2\times P}\right)}^{2}-\frac{1}{4D^{2}}}\right)$$

The formula calculates the central thickness of an IOL from variables normally supplied by manufacturers where T is the central thickness of IOL optic (mm); E is edge thickness of the IOL optic (mm); n2 is the refractive index of the IOL; P is IOL power (Diopter); and D is IOL optic diameter (mm).

For the type of IOL, we used E = 0.21 mm, n2 = 1.55, and D = 6 mm. *P*-values < 0.05 were considered statistically significant.

##  RESULTS

A total of 88 participants (115 eyes) fulfilled the inclusion criteria, among them 14 patients (18 eyes) were excluded due to low-quality scans, artifacts, decentration, and adhesion of posterior capsule to the IOL. Data from 97 eyes (74 patients) were used for the analyses. The patients included 30 men and 44 women with a mean age of 61.57 ± 8.08 years (range, 45–86 years). Known IOL power ranged from +11 to +35 D.

ICC (95% CI) for intra- and inter-observer repeatability was 0.999 (0.995–0.998) and 0.997 (0.996–0.998), respectively. IOL thickness correlated significantly with the labelled IOL power (R^2^ = 0.985, *P* < 0.001) [Figure 2]. The regression equation is as follows: IOL Power (D) = (0.04 × IOL thickness in micron) – 7.56. For instance, an IOL with a central thickness of 700 microns predicts an IOL power of 20.43D. Figure 3 shows a plot of labelled IOL power against IOL power calculated by the measured regression equation for our patients indicating a significant correlation (R^2^ = 0.970, *P* < 0.001). The majority of labelled IOL powers were within mean ± SD of difference of IOL power and calculated IOL power [Figure 4].

For each prediction, 95% CI width was generated (CI width = $\sqrt{0.695+\frac{{\left(IOLthickness\left(µ\right)-792.31\right)}^{2}}{627755}}$ ), yielding an average of 0.84 ± 0.02 (range, 0.83–0.91). For IOL thicknesses between 600 and 900 microns, 95% confidence interval did not exceed 0.86 D, however, farther from the mean, the CI was wider indicating that the accuracy of prediction is highest within this range and decreases with IOL thicknesses outside this range [Figure 5].

Central IOL thickness was also calculated theoretically using the Naeser et al formula.^[9]^ The calculated IOL thicknesses and the AS-OCT-measured central IOL thicknesses were significantly correlated (R^2^ = 0.984, *P* < 0.001).

##  DISCUSSION

In this study, we demonstrated that measuring the central thickness of SA60AT IOL by AS-OCT was highly repeatable and closely correlated with labelled IOL power, which could predict the IOL power within ± 0.85D of actual power. We also determined the correlation of AS-OCT-measured IOL thickness with the theoretically calculated IOL thickness and observed a significant correlation between the two thicknesses. Therefore, measuring IOL thickness using AS-OCT can provide an almost precise estimation of unknown IOL power using the Naeser et al formula as well.^[9]^ Although our results are most likely applicable to the specific IOL type and power range used in this study, many IOLs share the same characteristics in terms of size, optic diameter, and refractive index, so it is possible that the formula would apply to many IOL trademarks.

The use of accurate biometry techniques and appropriate IOL calculation formulas has greatly improved the refractive outcomes of cataract surgery. However, in cases with refractive surprise after cataract surgery, possible sources of error include decentered IOL, undiagnosed keratoconus, inaccurate biometry, upside down IOL implantation, incorrect IOL brought into the theatre, mixed-up documentation of patients, and mislabeled IOLs.^[3,4,5]^ In the past, some surgeons used to evaluate lens resolution and measure the IOL power before inserting the lens into the eye to make sure that the manufacturing IOL is truly the correct power.^[10]^

In a study by Turner et al using Scheimpflug imaging, the central thickness of MA60AC IOL (Alcon) was correlated with known IOL power and a similar formula was obtained. As compared to our study, they had a limited range of IOL power (11 to 26.5D).^[8]^ The IOL thickness measurements using Scheimpflug imaging was different from our AS-OCT-derived measurements, for example, a 600 micron thickness in their study represented IOL power of 20.451 D, while in our study 700 microns represent 20.43 D IOL power. The difference in measurements obtained by different imaging techniques and even among different OCT machines has been well-documented which indicates that the measurements cannot be used interchangeably.^[11,12,13]^

We observed that for IOL thicknesses between 600 and 900 microns, 95% confidence interval did not exceed 0.86 D, however, farther from the mean, CI was wider indicating that the accuracy of prediction is highest within this range and decreases with IOL thicknesses outside this range [Figure 5]. Additionally, IOL surprise most commonly happens in the extreme IOL powers with less accuracy in the prediction of the IOL power.

Our study had the advantage of including a wide range of IOL powers and comparing our measurements to the theoretic formula for calculating IOL thickness. However, using a single type of IOL precludes extrapolation of our results to other IOL brands with different designs or refractive index and powers outside the range used in this study. Since OCT imaging uses backscattered infrared light, for accurate IOL thickness measurements by OCT, media anterior to the IOL should be clear. Therefore, dense corneal opacities can interfere with image acquisition and accurate measurements, as are titling or decentration of the IOL, which preclude the presence and adjustment of reflex saturation beam as the indicator of perpendicularity of the IOL to the scanning beam. These limitations of IOL thickness measurement by OCT were considered as the exclusion criteria in our study. Attachment of posterior capsule to the IOL may result in falsely greater thickness. However, posterior capsule opacification (PCO) per se is not a limitation to IOL thickness measurements provided that the posterior capsule could be visualized separately behind the IOL.

In summary, our study was successful in determining that central IOL thickness measurements by AS-OCT shows a strong correlation with IOL power with high accuracy and repeatability and can be used with the regression equation obtained for this IOL type in cases of IOL surprise. Further studies with other types of IOLs are warranted to evaluate the applicability of our results. Future studies are needed to evaluate applicability of OCT to measure IOL tilt and the resulting induced cylinder. It is also recommended that the manufacturers provide IOL thickness on the IOL boxes along with other IOL characteristics, which could be compared with OCT-derived thicknesses in case of IOL surprise.

##  Financial Support and Sponsorship

Nil.

##  Conflicts of Interest

There are no conflicts of interest.References
